# Age-related differences in upper limb motor performance and intrinsic motivation during a virtual reality task

**DOI:** 10.1186/s12877-023-03970-7

**Published:** 2023-04-27

**Authors:** Ying Dong, Xiaoyu Liu, Min Tang, Hongqiang Huo, Duo Chen, Xin Du, Jinghui Wang, Zhili Tang, Xiaofeng Qiao, Jieyi Guo, Linyuan Fan, Yubo Fan

**Affiliations:** 1 Key Laboratory for Biomechanics and Mechanobiology of Ministry of Education, Beijing Advanced Innovation Center for Biomedical Engineering, School of Biological Science and Medical Engineering, Beihang University, Beijing, 100083 China; 2 State Key Laboratory of Virtual Reality Technology and Systems, Beihang University, Beijing, 100083 China; 3 School of Medical Science and Engineering Medicine, Beihang University, Beijing, 100083 China

**Keywords:** Virtual reality, Aging, Upper limb, Motor skill, Perceptive ability, Cognitive ability, Intrinsic motivation

## Abstract

**Background:**

In recent years, virtual reality (VR) has evolved from an alternative to a necessity in older adults for health, medical care, and social interaction. Upper limb (UL) motor skill, is an important ability in manipulating VR systems and represents the brain’s regulation of movements using the UL muscles. In this study, we used a haptic-feedback Virtual Box and Block Test (VBBT) system and an Intrinsic Motivation Inventory (IMI) to examine age-related differences in UL motor performance and intrinsic motivation in VR use. The findings will be helpful for the development of VR applications for older adults.

**Methods:**

In total, 48 young and 47 older volunteers participated in our study. The parameters including VBBT score, number of velocity peaks, velocity, grasping force and trajectory length were calculated to represent the task performance, manual dexterity, coordination, perceptive ability and cognitive ability in this study.

**Results:**

Age-related differences could be found in all the parameters (all *p* <  0.05) in VR use. Regression analysis revealed that the task performance of young adults was predicted by the velocity and trajectory length (R^2^ = 64.0%), while that of older adults was predicted by the number of velocity peaks (R^2^ = 65.6%). Additionally, the scores of understandability, relaxation and tiredness were significantly different between the two groups (all *p* <  0.05). In older adults, the understandability score showed large correlation with the IMI score (|r| = 0.576, *p* <  0.001). In young adults, the correlation was medium (|r| = 0.342, *p* = 0.017). No significant correlation was found between the IMI score and VBBT score (|*r*| = 0.142, *p* = 0.342) in older adults, while a medium correlation (|*r*| = 0.342, *p* = 0.017) was found in young adults.

**Conclusions:**

The findings demonstrated that decreased smoothness in motor skills dominated the poor VR manipulation in older adults. The experience of understandability is important for older adults’ intrinsic motivation in VR use.

## Background

Virtual reality (VR), as a kind of digital technology, is beginning to emerge for use in older adults [1, 2]. In recent years, VR has been used not only in commercial games for entertainment but also in serious games for health [3], medical care [4], and social interaction [5, 6]. The elderly in particular have benefited from this technology due to the outbreak of infectious diseases, such as COVID-19, since VR could be a helpful solution that meets requirements in health care due to isolation and protective measures [6, 7]. Recently, the use and interpretation of VR devices and tasks have evolved to be a necessity rather than an alternative. However, older adults commonly exhibit poor performance in VR interaction due to the decline in abilities related to motion, perception and cognition [8–10]. This tends to dampen their enthusiasm in VR participation. It has been reported that intrinsic motivation plays an important role in improving participants’ enthusiasm [11, 12]. Therefore, it is necessary to investigate the age-related differences in performance and intrinsic motivation in VR use for developing appropriate VR systems for older adults.

Upper limb (UL) motor skill is an important ability representing the brain’s regulation of movements using the muscles of the hands, wrists, elbows and shoulders [13]. Older adults exhibit an evident decline in UL motor performance because of remodeled or atrophied muscle fibers [14, 15], weakened sensitivity of tactile and kinesthetic receptors [16], reduced speed of peripheral nerve conduction [17, 18] and deteriorated structure and function in motor-related brain regions [19]. These retrogressive changes may lead to low smoothness [20, 21] and speed [20, 22] of movements, inappropriate grasping forces regulated by haptic perception (mediated by cutaneous and kinesthetic) [9, 23] and unoptimized routes in task execution related to cognitive ability [24]. These changes can be characterized by kinematic or kinetic parameters [25–27]. However, few studies have indicated the differences in contributions of those parameters between young and older adults in VR performance. A meta-analytic review suggested that intrinsic motivation could promote engagement in an activity via the internal satisfaction caused by the enjoyment and quality of the experience [28]. Motivation might be stimulated by the game itself or by the immersive quality of VR technology [29]. To the best of our knowledge, few studies have explored the motivational affordances of VR use in older adults. The understanding of factors related to intrinsic motivation in VR use is important to provide preliminary data to guide the development of VR applications for older adults.

Haptic immersion, an important element in VR technology, provides the perception of texture, weight and compliance of manipulated objects, allowing users to interact with virtual environments in a more realistic manner [26]. Kinematic and kinetic measures obtained by haptic devices are validated to quantify users’ performance. Previous studies reported that VR systems with haptic devices can be used to identify the impairments of patients with deficiencies in UL motor function [30–32]. The Box and Block Test (BBT) has been widely used to assess UL motor ability due to its merits, such as simple operation, short time consumption and high validity [33, 34]. In our previous study, we developed a virtual box and block test (VBBT) system to examine the task performance of stroke patients and found that the kinematic and kinetic metrics obtained from haptic devices were effective in characterizing their motor functions [35].

In the current study, we used the VBBT system to interpret 1) the differences in motor, perceptive and cognitive abilities between older and young adults during VR use; 2) the weight of motor, perceptive and cognitive abilities in the contribution to VR performance of older and young adults; and 3) the difference in intrinsic motivation toward VR use between older and young adults. The hypothesis was that there were significant differences in UL motor performance and intrinsic motivation in VR use between young and older adults. The findings will be helpful for the development of VR applications for older adults.

## Methods

### Ethical approvals

The current study adhered to the tenets of the Declaration of Helsinki, and ethical approval was obtained from the Biological and Medical Ethics Committee of Beihang University (Number: BM20180017). Each participant was given written and verbal information on the current study, and written informed consent was obtained prior to study involvement.

### Participants

Forty-eight young volunteers (age: mean ± SD = 28.03 ± 7.07 years, range = 18–45 years; 28 females and 20 males) and 47 older volunteers (age: 71.09 ± 7.05 years; 60–87 years; 34 females and 13 males) were enrolled in this study. All participants were right-handed with normal or corrected-to-normal vision and without any neurological disorder, musculoskeletal impairment or cyber sickness. Older participants were excluded if they were incapable of normal cognitive function as assessed by the Mini-Mental State Evaluation score (MMSE < 24).

### Experimental setup

**Fig. 1:**
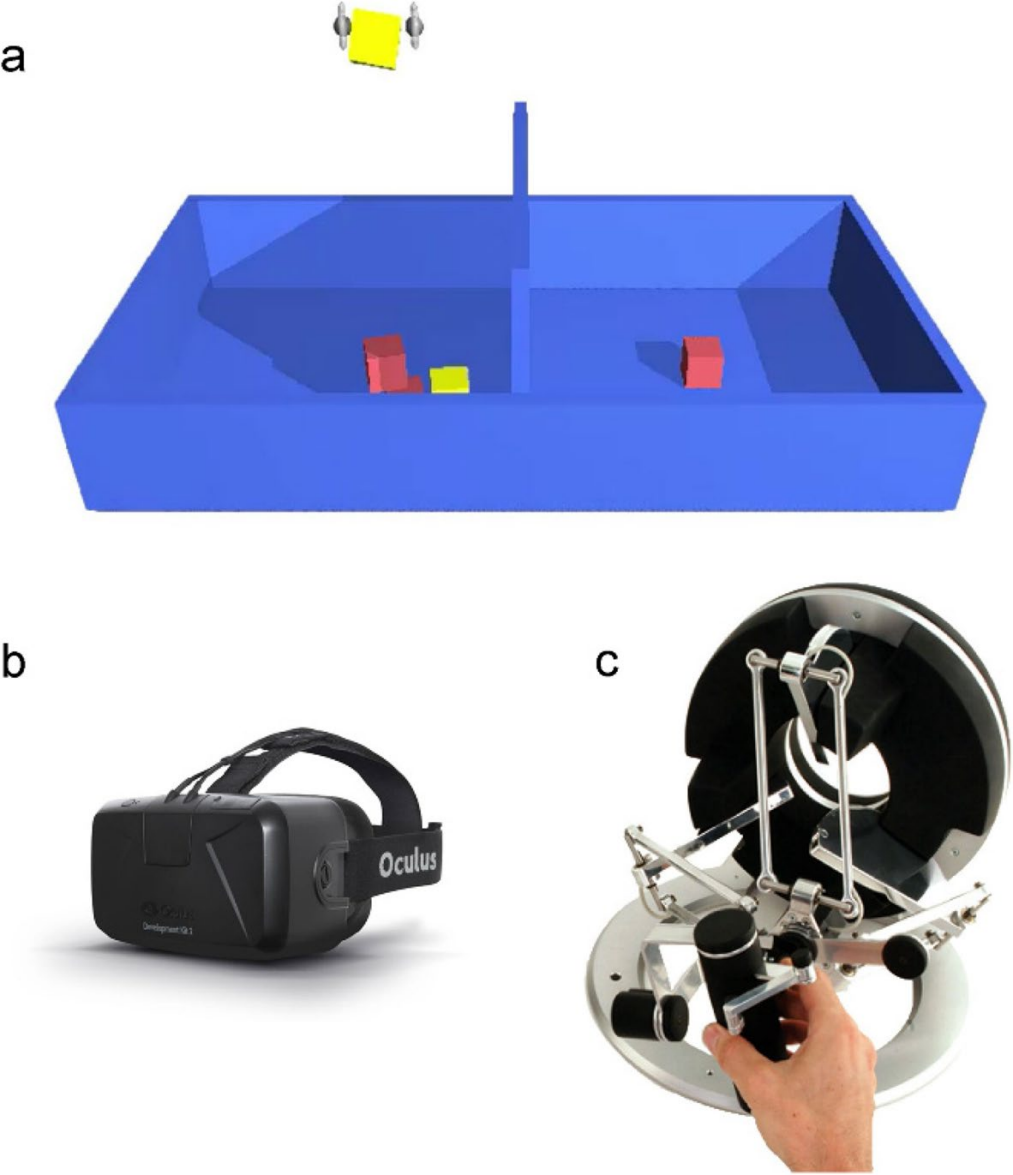
The VBBT system. **a** The VBBT scenario. **b** The VR headset. **c** The haptic feedback device

The experimental setup has been reported in our previous study [35]. In the VBBT scenario, a virtual test box with a barrier partition in the middle was created in the VR environment (Fig. 1a). The VBBT system consisted of a VR headset (Oculus Rift, Facebook Inc., U.S.; Fig. 1b), which was used to provide a 3D virtual environment, as well as a haptic device (Omega.7, Force Dimension Inc., Switzerland; Fig. 1c), which was used to provide haptic feedback to the participant’s hand and precisely collect the movement data. The handle of the haptic device is represented by a virtual grasping tool. As a participant operated the handle, the grasping tool was synchronously operated in the virtual environment. The force threshold was set to 0.2 N. The block would drop if the grasping force was under the threshold. At the beginning of the VBBT, there was one block that was created in the compartment of the box on the side of the tested hand. The virtual box and each block were attributed physical properties, including tactile contact and gravity (block: 8.82 × 10^− 2^ Newtons). During the VBBT performance, when a participant had completed one trial in which a block was moved from one compartment to the other, another block was then automatically created.

### Experimental procedure

**Fig. 2:**
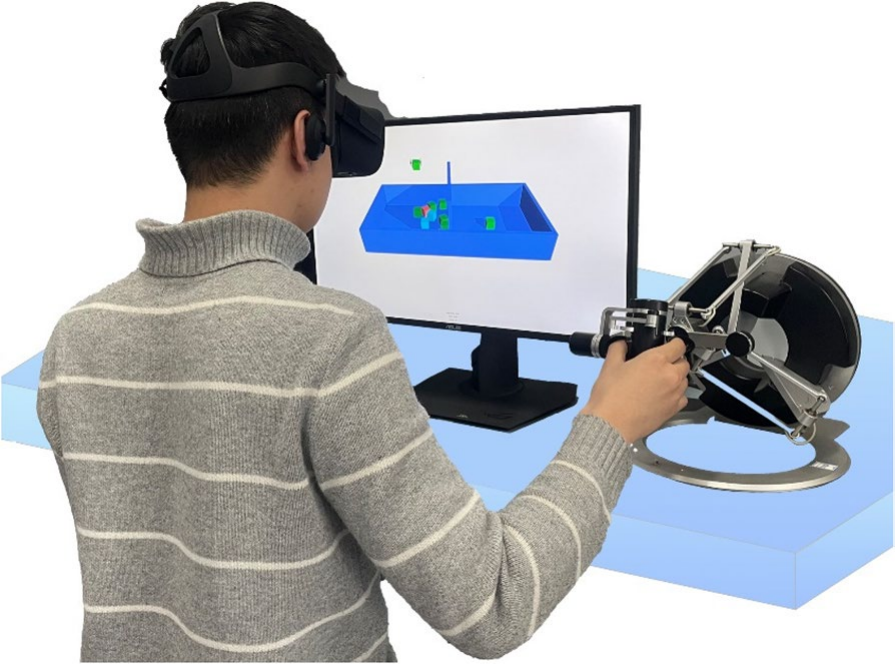
Participant manipulated the VBBT system

The participants were seated on a standard height chair with their left hand pronated and rested on a table on their left side, with the right elbow flexed approximately 90 degrees and the shoulder abducted approximately 30 degrees. The haptic device was placed on the table before the participants (see Fig. 2). We first instructed the participants on how to operate the haptic device. Then, the participants, wearing the VR headset, were given a familiar session before the formal test. In the familiar session, there was enough time for the participants to manipulate the VBBT system until they thought they were sufficiently comfortable with it and were capable of moving the blocks as fast as possible. In the formal tests, the participants were given 1 min to move as many blocks as they could until the program automatically stopped.

**Fig. 3:**
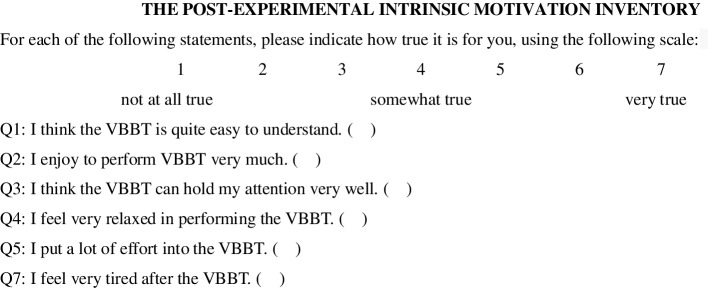
The Intrinsic Motivation Inventory for all participants

After the participants finished the VBBT, they were given a simplified Intrinsic Motivation Inventory (IMI, Fig. 3) to evaluate their experiences of the VR use, and an informal interview was conducted regarding their experiences. In the IMI, there were 6 sentences corresponding to 6 items, including difference, understandability, enjoyment, attraction, relaxation, effort and tiredness. The IMI score was the sum of each item score except for effort and tiredness, the scores of which should be subtracted from 8 for this method could indicate more of the concept described for intrinsic motivation.

### Measurement

In the VBBT, some specific parameters were computed to investigate the motor, perceptive and cognitive abilities of the participants. Originally, to the number of moved blocks, 3D position and velocity of the virtual block, as well as the grasping force, were collected by the haptic device. The number of moved blocks in 1 min was referred to VBBT score. All signals were sampled at a frequency of 100 Hz and were stored on a computer (IntelCore 7, 3.2 GHz, Windows 10). Then, a 2nd-order lowpass Butterworth filter with a cut-off frequency of 6 Hz was used to filter the data. All the parameters were computed by the mean value across all the moved blocks for each participant. For the purpose of our research, in addition to the VBBT score, the kinematic and kinetic parameters we computed in the VBBT were defined as follows.

#### Number of velocity peaks

The number of velocity peaks in a virtual block transfer, provided an estimation of the number of submovements that represented repetitive accelerations and decelerations for completing the movement segment [30, 36]. In our study, it was a measure of movement smoothness and UL coordination that would affect the accuracy of VR manipulation [37]. The lower the number of velocity peaks was, the better the movement smoothness of the VR manipulation.

#### Velocity

The mean value of the velocity in a virtual block transfer, was used to evaluate movement speed [38, 39]. In our study, it was a measure of manual dexterity affecting the efficiency of VR manipulation. The higher the velocity was, the better the manual dexterity of the VR manipulating. The velocity value was calculated using Eq. 1 for statistical analyses

1 $$V=\frac{\sum_{i=1}^n\sqrt{\ {V}_{x,i}^2+{V}_{y,i}^2+{V}_{z,i}^2}\ }{n}$$

where *n* is the number of sampling points; *V* is the mean value of the velocity in a virtual block transfer; and *V*_*x*, *i*_, *V*_*y*, *i*_, and *V*_*z*, *i*_ are the velocities along the x-axis, y-axis and z-axis, respectively, collected by the haptic device.

#### Grasping force

The mean of the grasping force in a virtual block transfer, was used to indicate how much effort the participant used to overcome resistance and make an object move during the transferring task. In our study, it was a performance that reflected the perceptive ability to perceive the weight, texture and compliance of the virtual object [40] in VR manipulation. The larger the grasping force was, the lower the haptic perception ability. The value of grasping force was calculated using Eq. 2 for statistical analyses

2 $$F=\frac{\sum_{i=1}^n{F}_i\ }{n}$$

where *n* is the number of sampling points, *F* is the mean value of the grasping force in a virtual block transfer, and *F*_*i*_ is the grasping force collected by the haptic device.

#### Trajectory length

The length of the actual trajectory in a block transfer trial, reflected the task optimization ability [41]. In our study, it was a performance to represent the cognitive abilities of motor planning and executive ability in the VR task. The shorter the trajectory length was, the better the cognitive ability. The value of trajectory length was calculated using Eq. 3 for statistical analyses

3 $$\textrm{S}=\sum\nolimits_{i=1}^{n-1}\sqrt{{\left({x}_{i+1}-{x}_i\right)}^2+{\left({y}_{i+1}-{y}_i\right)}^2+{\left({z}_{i+1}-{z}_i\right)}^2}$$

where *n* is the number of sampling points; *S* is the mean value of the trajectory length in a virtual block transfer; and *x*_*i*_, *y*_*i*_, and *z*_*i*_ are the position coordinates on the x-axis, y-axis and z-axis, respectively, collected by the haptic device.

#### IMI

The IMI is a measurement instrument that is intended to assess participants’ subjective experience related to a target activity in laboratory experiments [42]. In our study, it was used to assess the understandability, enjoyment, attraction, relaxation, effort and tiredness of each participant during the VBBT. A higher score for each aspect indicated that the participant experienced more of the indicated aspect, except for the effort and tiredness, because these two scores are the reverse of the participant’s response concerning intrinsic motivation.

### Statistical analysis

We calculated the mean and variability (i.e., standard deviation: SD) of each parameter produced by each trial. All data were analyzed using SPSS 23.0 (Statistical Package for Social Sciences Inc. Chicago, IL, USA). The normality of the parameters was tested using histogram plots and Shapiro–Wilk tests. Independent sample t tests were performed to compare age-related differences in parameters of velocity, VBBT score and trajectory length between young and older adults because the data were normally distributed. The Mann–Whitney nonparametric U test was performed to compare age-related differences in the parameters number of velocity peaks and grasping force between the two groups due to nonnormal distributions. In each group, we conducted a stepwise multiple linear regression analysis to determine which parameters could predict task performance in each group. These analyses were performed to investigate whether the predictors of task performance were similar in each group. The group (young and older adults) was created as a dummy variable and used as a moderating variable in the regression analysis to determine the differences in contributions of kinematic and kinetic parameters in VR performance between young and older adults. Pearson (if the distributions of the variables were normal) and Spearman’s rank correlation coefficients (if the distributions of the variables were abnormal) were used to determine the correlation between pairs of all independent variables, and those with correlation coefficients greater than 0.7 were not included in the same model [43]. The analysis of the IMI scores for the VR use between the two groups was performed by nonparametric tests since they were ordinal variables [44]. Spearman’s rank correlation coefficients were computed among each item score and IMI score and the VBBT score. Correlations were considered trivial (*r* <  0.1), small (0.1 ≤ *r* <  0.3), medium (0.3 ≤ *r* <  0.5) and large (*r* > 0.5) according to Cohen’s conventions [45].

## Results

### Group differences in measures

Table 1 shows that all the parameters, including the VBBT score, velocity, number of velocity peaks, grasping force and trajectory length were significantly different between older and young adults (all *p* <  0.001). It indicated thatnparticipants’ VR performance, movement smoothness and speed, haptic perception as well as motor planning and executive abilities in VR use was worse than those of young adults.

**Table 1:** Differences in each parameter between young and older adults

Parameters	Older adults Mean (SD)	Young adults Mean (SD)	***p***	Cohen’s d
**VBBT score**	21.85 (5.53)	35.88 (5.27)	< 0.001	2.60
**Number of velocity peaks**	4.92 (1.99)	2.56 (0.55)	< 0.001	−1.62
**Velocity (m/s)**	0.14 (0.04)	0.20 (0.03)	< 0.001	1.70
**Grasping force (N)**	4.20 (0.59)	3.69 (0.67)	< 0.001	−0.81
**Trajectory length (mm)**	265.82 (33.17)	233.90 (21.90)	< 0.001	−1.14

**Fig. 4:**
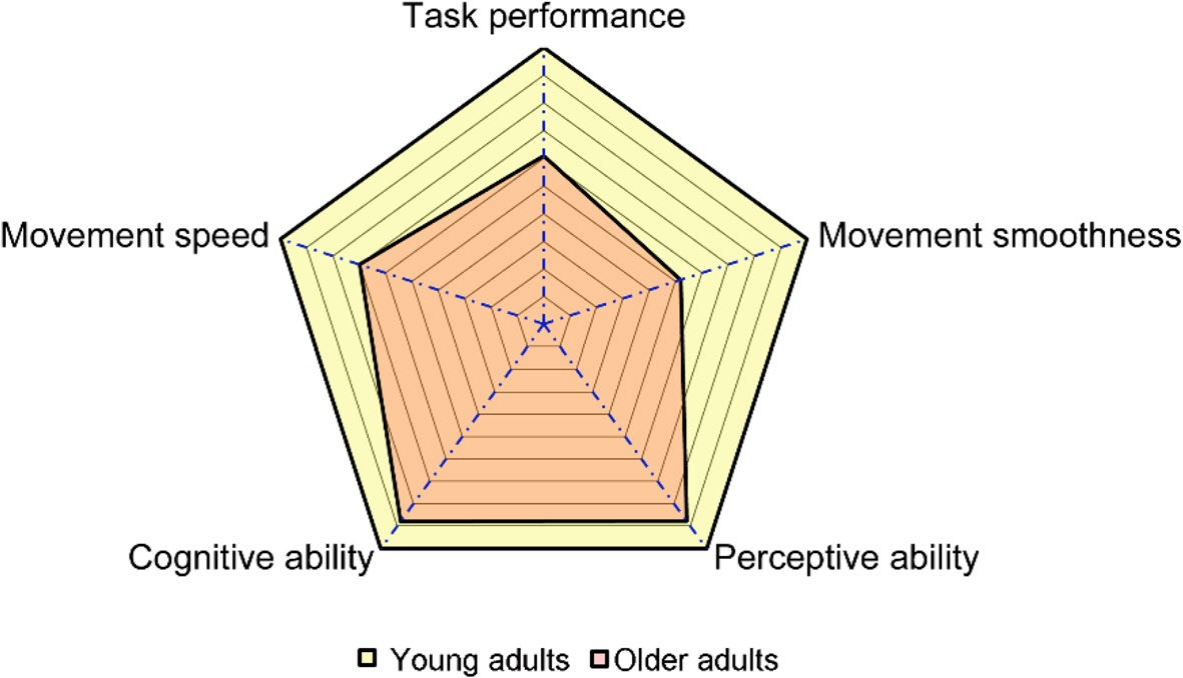
Radar chart for older and young adults

A radar chart (see Fig. 4) was plotted to show the differences in abilities including the task performance, movement speed, movement smoothness, cognitive ability and perceptive ability between older and young adults. In the chart, the values of parameters in both older and young adults were normalized by the relative value of young adults, i.e., values of the parameters in both groups were divided by the relative values of the young group. If a value for older adults was larger than that for young adults, then its reciprocal was calculated.

### Models of multiple linear regressions in older and young adults

Multiple linear regression was conducted to predict participants’ task performance with the VBBT score from the kinematic and kinetic parameters that represented the motor, perceptive, and cognitive abilities. In older adults, the prediction model of task performance was explained by the number of velocity peaks, F (45, 1) = 88.55, *p* < 0.001. The beta weight of the number of velocity peaks was − 0.814 (see Table 2).

**Table 2:** Model of VBBT score by kinematic and kinetic parameters in older adults

Parameters	Adjusted R^**2**^	F	***p***	β	VIF
**VBBT score**	0.656	88.55	< 0.001		
**Number of velocity peaks**				−0.814	1.000

In young adults, the prediction model of task performance was explained by the parameters of velocity and trajectory length, F (45, 2) = 42.87, *p* < 0.001. The beta weights of velocity and trajectory length were 0.797 and − 0.326, respectively (see Table 3).

**Table 3:** Model of VBBT score by kinematic and kinetic parameters in young adults

Parameters	Adjusted R^**2**^	F	***p***	β	VIF
**VBBT score**	0.640	42.81	< 0.001		
**Velocity**				0.797	1.028
**Trajectory length**				−0.326	1.028

Group was one of the predictors of VBBT score (see Table 4). This results indicated that the contributions of kinematic and kinetic parameters between young and older adults in VR performance is significantly different.

**Table 4:** Model of VBBT score by kinematic and kinetic parameters in young and older adults

Parameters	Adjusted R^**2**^	F	***p***	β	VIF
**VBBT score**	0.888	249.01	< 0.001		
**Velocity**			< 0.001	0.627	1.754
**Trajectory length**			< 0.001	−0.300	1.361
**Group**			< 0.001	0.242	2.219

### Group differences in each item score and IMI score

Table 5 shows that the scores of items, including understandability (*p* = 0.021), relaxation (*p* = 0.031) and tiredness (*p* = 0.046) in older adults were significantly different than those in young adults. It indicated that although older adults could not understand the VBBT task as well as young adults, they felt more relaxed and less exhausted during the VBBT task. No significant difference was found between older adults and young adults in the scores of other items, including enjoyment, attraction and effort. There was no significant difference in the IMI score between the two groups.

**Table 5:** Differences in each item score and IMI score between young and older adults

Items	Older adults Mean (SD)	Young adults Mean (SD)	***p***
**Understandability**	6.13 (1.44)	6.73 (0.64)	**0.021**
**Enjoyment**	6.40 (0.92)	6.19 (1.00)	0.214
**Attraction**	6.62 (1.11)	6.56 (0.99)	0.223
**Relaxation**	6.32 (1.35)	5.66 (1.77)	**0.031**
**Effort**	2.11 (1.81)	1.96 (1.13)	0.393
**Tiredness**	1.94 (1.81)	2.21 (1.47)	**0.046**
**IMI score**	37.43 (4.77)	36.96 (3.86)	0.280

### Correlational results among the score of each item, IMI score and VBBT score

Table 6 shows the correlations between each item score and IMI score in each group. Besides the scores of relaxation, effort and tiredness items (|*r*| = 0.508 to 0.649, all *p* < 0.001), the score of understandability item showed large correlations with the IMI score (|*r*| = 0.576, *p* < 0.001) in older adults. While in young adults, the score of enjoyment item showed large correlations with the IMI score (|*r*| = 0.520, *p* < 0.001).

**Table 6:** Correlations between each item score and IMI score in each group

	Understandability	Enjoyment	Attraction	Relaxation	Effort	Tiredness
**Older adults IMI score**	0.576^**^	0.348^*^	0.135	0.508^**^	−0.562^**^	− 0.649^**^
**Young adults IMI score**	0.342^*^	0.520^**^	0.302^*^	0.661^**^	−0.568^**^	−0.724^**^

^*^Significant correlation *p* < 0.05

^**^Significant correlation *p* < 0.001

Table 7 shows the correlations between each item score and the VBBT score in each group. No significant correlation was found between each item score and the VBBT score (|*r*| = 0.046 to 0.268, *p* = 0.069 to 0.761) in older adults. In young adults, no significant correlation was found between each item score and the VBBT score (|*r*| = 0.042 to 0.197, *p* = 0.180 to 0.777), except for the enjoyment score, which showed a medium correlation (|*r|* = 0.435, *p* = 0.002) with the VBBT score.

**Table 7:** Correlations between each item score and the VBBT score

	Understandability	Enjoyment	Attraction	Relaxation	Effort	Tiredness
**Older adults VBBT score**	0.268	0.265	0.046	−0.087	−0.134	− 0.169
**Young adults VBBT score**	0.125	0.435^**^	−0.129	0.195	−0.042	−0.197

^*^Significant correlation *p* < 0.05

^**^Significant correlation *p* < 0.001

**Fig. 5:**
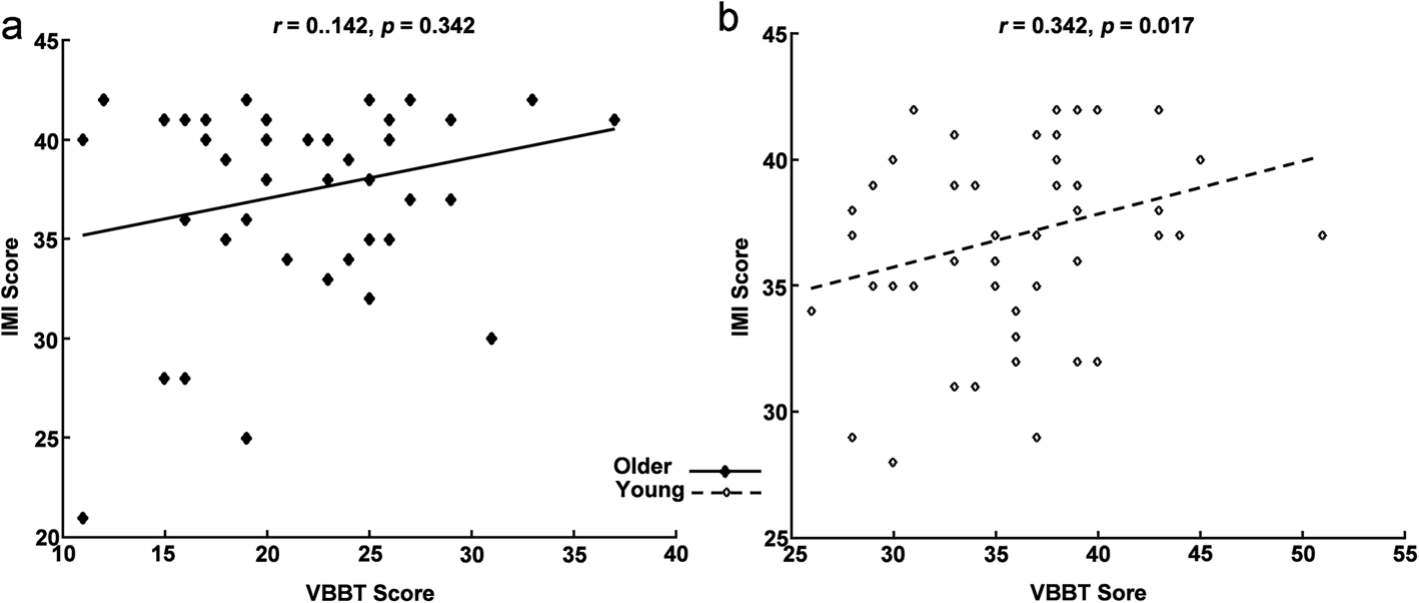
Correlations between IMI score and VBBT score. **a** Correlations between IMI score and VBBT score in older adults. **b** Correlations between IMI score and VBBT score in young adults

Figure 5 shows the correlations between the IMI score and the VBBT score in each group. No significant correlation was found between the IMI score and VBBT score (|*r*| = 0.142, *p* = 0.342) in older adults, while a medium correlation was found between the IMI score and VBBT score (|*r*| = 0.342, *p* = 0.017) in young adults.

## Discussion

In the current study, we used the VBBT system to examine the differences in task performance, motor, perceptive and cognitive abilities and intrinsic motivation in VR use between older and young adults. We determined kinematic and kinetic parameters that could be used to predict task performance and reflect the variance in VBBT operation. Additionally, we compared IMI scores between the two groups to assess their intrinsic motivation. Our results were expected to help the design of VR devices for older adults in the future.

In recent years, the combination of VR technology and haptic devices has been used to provide a high degree of controlled and manipulated stimuli, allowing various customization for various UL tasks [46]. Haptic perception refers to active manual exploration accompanied by afferent sensation that is based on the cumulative neural input from mechanoreceptors (articular, muscular, and cutaneous receptors) [23, 47, 48]. The density of mechanoreceptors decreases, nerve conduction velocity and sensory nerve action potentials slowdown in old age [49–51]. Decreased and diminished signals experienced by older adults experienced are important for signaling object friction, object slippage, and grasp force magnitude [52, 53]. A haptic-feedback system was used to provide sensory information about the size, texture and stiffness of the virtual object as well as to simulate the feeling of grasping in our study. A larger grasping force in older adults was considered to reflect a motor strategy that compensated for changes in haptic perception because a larger grasp force may secure virtual objects in people’s control for a wide range. In this case, the dexterity and manual speed in VR manipulation may be compromised due to the increased muscle activation levels required to produce the additional force [54]. Furthermore, excessive force will have the further effect of reducing smoothness in motor control [55, 56]. Previous empirical researches have demonstrated decreases in prefrontal cortex gray matter volume [57, 58], deteriorations in frontal and parietal white matter [59, 60] and reduced levels of neurotransmitters [61–63] in older adults which lead to a decline in cognitive skills. a previous study reported that trajectory length is the only kinematic parameter that can reflect cognitive abilities, including motor planning and executive abilities [64]. Longer trajectories represent less precise movement to the target [41]. This suggests that control of precision during VR manipulation should be considered at the level of cognitive decline in older adults.

Regression results revealed that the performance in VR manipulation was predicted by the velocity and trajectory length, accounting for 64.0% of the variance in the VBBT score among young adults. While 65.6% of the variance in the VBBT score was significantly predicted by the number of velocity peaks in older adults. This suggested that VR use in older adults was mainly associated with the movement smoothness reflected by the number of velocity peaks, which may be caused by the increased noises in movement execution leading to increased submovements [65–68] in the motor output stage. While in young adults, performance in VR use involves both motor skills and specific cognitive abilities, such as the optimized trajectory ability [41, 69]. Therefore, the decreased movement smoothness in older adults is a critical obstacle for VR manipulation. It demonstrated that movement smoothness should be taken into consideration when VR systems were designed for the elderly.

High IMI scores were found in both young and older adults, which might be due to the characteristics of VR [70]. A head-mounted display, for example, was experienced as particularly motivating for older adults. Differences in each item and total IMI scores were significant in older and young adults. Compared to young adults, a lower score on the understandability item was found in older adults. The results of the informal interviews showed that older adults who were seldom exposed to VR in their daily life were unable to easily understand the VR task, while most young adults experienced VR use more frequently than older adults. It was unexpected that older participants felt more relaxed and less tired than the young participants, although older participants produced larger forces for grasping and longer trajectories for block movement. Such experiences can be explained by researchers that a higher interest makes activities feel relatively tireless and relaxing regardless of much effort [71–73]. Furthermore, the correlation analysis revealed that understandability was an important experience for high intrinsic motivation in older adults, compared to young adults who regarded enjoyment as a more important motivation. It suggested that VR systems specified for older adults should be easy to understand. The VBBT score could be regarded as a utilitarian index, which was defined in the literature [74]. In line with previous findings [75], we found that older adults prioritized intrinsic motivation (e.g., quality of experience) in the VR use while the utilitarian index was more important for young adults.

Our study presented the differences in motor, perceptive and cognitive abilities as well as intrinsic motivation in VR use between older and young adults. These findings will be helpuful to determine what should be considered when designing VR systems for older adults. However, several limitations of this study should be addressed. First, we did not recruit older adults with cognitive impairment or frailty. With the aging population, the prevalence of such older adults is increasing [76, 77]. We will recruit older adults with cognitive impairment or frailty in a future study. Another limitation is that we only used the VBBT to evaluate the performance of VR use. The VBBT was designed based on the BBT, which is a classic assessment for manual dexterity. We plan to examine the performance of older adults in VR use based on more varied VR scenarios and systems.

## Conclusions

This study showed differences in task performance, motor, perceptive and cognitive abilities as well as intrinsic motivation for VR use between older and young adults. The findings demonstrated that movement smoothness in motor skills was the predictor of VR performance in older adults, while in young adults, movement speed, motor plan and executive abilities were the main predictors. Understandability played an important role in the intrinsic motivation of older adults for VR use, while for young adults, enjoyment was important for the intrinsic motivation. This finding demonstrated that when developing VR applications for older adults, age-related differences in upper limb motor performance and intrinsic motivation in VR use should be taken into consideration.

## Data Availability

All the data and materials in the current study are available from the corresponding author on reasonable request.

## Abbreviations

VR: Virtual reality
VBBT: Virtual box and block test
BBT: Box and block test
IMI: Intrinsic motivation inventory
MMSE: Mini-Mental State Evaluation
SPSS: Statistical Package for Social Sciences
SD: Standard deviation
VIF: Variance inflation factor
